# Unraveling the Structural, Dielectric, Magnetic, and Optical Characteristics of Nanostructured La_2_NiMnO_6_ Double Perovskites

**DOI:** 10.3390/nano12060979

**Published:** 2022-03-16

**Authors:** Kang Yi, Qingkai Tang, Zhiwei Wu, Xinhua Zhu

**Affiliations:** National Laboratory of Solid State Microstructures, School of Physics, Nanjing University, Nanjing 210093, China; 15255458807@163.com (K.Y.); tangqinkai@126.com (Q.T.); mg20220132@smail.nju.edu (Z.W.)

**Keywords:** La_2_NiMnO_6_ double perovskites, nanoparticles and nanorods, hydrothermal process, physical properties, structural characterization

## Abstract

Double perovskite La_2_NiMnO_6_ (LNMO) nanoparticles and nanorods were synthesized via a hydrothermal process, where only aqueous inorganic solvents are used to regulate the microscopic morphology of the products without using any organic template. They crystallized in a monoclinic (*P*2_1_/*n*) double perovskite crystal structure. The LNMO nanoparticles exhibited spherical morphology with an average particle size of 260 ± 60 nm, and the LNMO nanorods had diameters of 430 ± 120 nm and length about 2.05 ± 0.65 μm. Dual chemical oxidation states of the Ni and Mn ions were confirmed in the LNMO samples by X-ray photoelectron spectroscopy. Strong frequency dispersion dielectric behavior observed in the LNMO ceramics, is attributed to the space charge polarization and the oxygen vacancy induced dielectric relaxation. A ferroelectric—paraelectric phase transition appearing near 262 K (or 260 K) in the LNMO ceramics prepared from nanoparticles (or nanorods) was identified to be a second-order phase transition. The LNMO samples are ferromagnetic at 5 K but paramagnetic at 300 K. The LNMO nanoparticles had larger saturation magnetization (*M*_S_ = 6.20 *μ*_B_/f.u. @ 5 K) than the LNMO nanorods (*M*_S_ = 5.68 *μ*_B_/f.u.) due to a lower structural disorder in the LNMO nanorods. The semiconducting nature of the nanostructured LNMO with an optical band gap of 0.99 eV was revealed by the UV–visible absorption spectra. The present results enable the nanostructured LNMO to be a promising candidate for practical spintronic devices.

## 1. Introduction

In recent years, double perovskites (DPs) R_2_NiMnO_6_ (R represents a rare earth element) have been widely investigated due to their rich multifunctional properties, such as ferroelectricity, ferromagnetism as well as magneto-capacitance (MC), which make them potential candidates for memory devices, sensors, and spintronic devices [1,2,3]. Their intriguing physical properties originate from the interactions between the B-sited strongly correlated 3d-transitional Ni and Mn ions [4,5,6]. It is reported that the size, charge, valence state, and cation ordering of the B-sited ions play a central role in modifying the physical properties of the R_2_NiMnO_6_ family [7,8,9,10]. Much effort has been deviated to improving their magnetocaloric and MC properties for novel device applications [11,12,13,14]. As one member of the R_2_NiMnO_6_ family, La_2_NiMnO_6_ (LNMO) is reported to be a ferromagnetic (FM) insulator with a Curie temperature (*T*_C_) of 280 K [7,15]. Its FM ordering is controlled by the 180° super-exchange interaction between the Ni^2+^-O^2−^-Mn^4+^ pair [16,17]. LNMO crystallizes in a monoclinic (*P*2_1_/*n*) structure in low temperature and a rhombohedral structure (*R*$\bar{3}$) in high temperature [3,15]. However, both of them could co-exist in a large temperature range, covering room temperature [18]. The saturation magnetization (*M*_S_), *T*_C_, and evolution of other phases like spin-glass in the LNMO are dependent upon the cation ordering and cation valences, which are significantly influenced by the synthesized conditions [19,20,21]. Actually, in the published literature different temperature dependent magnetic properties of the LNMO under field-cooled (FC) and zero-field cooled (ZFC) modes are reported by several research groups [22,23,24,25]. These discrepancies may have arisen from various synthesized routes used for the LNMO samples, which affect significantly the crystal structures and the extent of the anti-site defects (ASD) at Ni/Mn sites. That plays a vital role in determining the multifunctional properties (e.g., Curie temperature, optical band gap, dielectric tunability) of the LNMO samples. Furthermore, some conflicting viewpoints are also found in the literature with respect to the magnetic phase transitions and chemical valence states of the B-sited magnetic ions (e.g., Mn and Ni) as well as the magnetically ordered states [26]. For instance, magnetic transition at low temperature (~150 K) was reported in an epitaxial LNMO thin film, which was ascribed to the disordered phase [25]. However, this low temperature magnetic transition in single phase LNMO prepared via the Pechini method was attributed to the ASD that led to the FM-spin glass transition [13]. Yuan et al. [27] assigned this low temperature magnetic transition of the LNMO ceramics to the FM interactions between the Ni^3+^ and Mn^3+^ Jahn-Teller ions. The high *T*_C_~280 K of LNMO with an ideal ordered rock salt structure was first reported, which was attributed to the 180° superexchange interaction between the Ni^2+^ − O^2−^ − Mn^4+^ pair [27]. However, other chemical valence states, such as Mn^3+^ and Ni^3+^ could be stabilized depending upon the synthesized conditions, and their superexchange interactions also led to the FM ordering [28]. As a consequence, two kinds of FM transitions around temperatures of 266 K and 100 K were reported in the LNMO system [15,25]. The lower temperature FM transition was ascribed to the non-uniformities of the samples where two phases (Ni^2+^-Mn^4+^ and Ni^3+^-Mn^3+^) co-exist, exhibiting many different electronic properties [15,22,25]. While Choudhury et al. [13] assigned this lower temperature FM transition (observed in partially disordered LNMO) to result from the magnetic frustration in the system due to the presence of ASD, which led to strong AFM interactions via the Ni^2+^-O^2−^-Ni^2+^ and Mn^4+^-O^2−^-Mn^4+^ bonds. Interestingly, the LNMO nanoparticles (NPs) with an orthorhombic crystal structure (space group Pbnm), exhibited only one magnetic transition around 200 K [29]. In addition, relaxor-like ferroelectricity and multiferroic behaviors were reported in the sol-gel derived LNMO NPs [30], but in the polycrystalline LNMO samples prepared by the sol-gel method, the relaxor-like dielectric behavior was completely excluded by Chandrasekhar et al. [10].

Over the past decade, LNMO has been prepared in the forms of bulks, thin films, NPs and/or nanorods (NRs) [31]. Numerous works have been performed to resolve the issues of local valance states, ASD, anti-phase boundaries induced physical properties, and the nature of the short-range magnetic correlation above *T*_C_ in the LNMO system [4,6,26]. It is widely accepted that the physical properties are strongly influenced by B-site cation disordering, which has a strong correlation with the selection of synthesis routes [10]. Up to date, various synthetic methods have been developed to synthesize nanostructured LNMO materials, such as but not limited to the solid-state reaction method [3,10,32,33,34], sol-gel process [20,29,35,36,37], gel combustion method [38,39], co-precipitation method [40,41], Pechini method [9,15], molten salt method [42,43,44], ionic coordination reaction method [45], and hydrothermal method [46,47]. Despite the sol-gel process being commonly used in the literature for the synthesis of LNMO NPs, this technique often suffers from being out of control over morphology, and yields poorly-defined NPs. Similarly, NPs synthesized by the molten salt synthesis method often have a tendency to produce poly-dispersed particles in terms of both size and shape. In contrast, highly uniform LNMO NRs with an average diameter of ∼20 nm and length of ~120 nm (aspect ratio = 6.0) were synthesized by using a two-step hydrothermal procedure, where the organic surfactant cetyltrimethylammonium bromide (CTAB) was used as a capping agent [46]. Very recently, both LNMO nanocubes with sizes in the range of ~70 to 400 nm and LNMO NRs with average diameters of ~50 nm were synthesized by using a surfactant-less hydrothermal method [47]. It is found that the LNMO NRs have a 1.5-fold increase in the methanol oxidation reaction activity compared to LNMO NPs. However, currently, only a few works on the hydrothermal synthesis of nanostructured LNMO are reported [46,47]. In parallel with the experimental investigations of LNMO DP oxide, theoretical calculations by using the Density Functional Theory (DFT) are also performed to understand the structural, electronic, and magnetic properties of the LNMO [48,49]. In addition, theoretical simulations by DFT calculations also assist the understanding of the structure-property relations for complex nanostructured systems of similar nature and a similar level of complexity (e.g., containing transition metal atoms as well as a variety of lighter elements), which are directly supportive to the credibility of the physical mechanisms involved in complex nanostructured systems [50,51,52]. 

Overall, to gain the full understanding of the structural, dielectric, magnetic, and optical properties of the LNMO system more systematic studies are required. The hydrothermal process has the advantages of controllable sizes and morphologies of the final products, which are strongly dependent upon the starting precursors, reaction temperature and time, pH value, the types and concentrations of mineralizers. In this work, we choose the hydrothermal process to synthesize the LNMO NPs and NRs without using any surfactant to assist the formation of the LNMO NRs, instead, we only adjust the concentration of NaOH to control the microscopic morphologies of the obtained products. In contrast, in the previous work, Gaikwad et al. [46] used the organic surfactant CTAB as a capping agent to synthesize the LNMO NRs with an average diameter of ∼20 nm and length of ~120 nm (aspect ratio = 6.0). In contrast, our LNMO NRs had an average diameter of 430 ± 120 nm and length about 2.05 ± 0.65 μm, and their aspect ratio was as high as 13.0. As compared with the previously reported hydrothermal method for the synthesis of LNMO nanomaterials, our present work completely uses the aqueous inorganic solvents and does not utilize any organic template to regulate the microscopic morphology of the products, so the synthesis method is much simple and non-toxic. In addition, the B-site ordering degrees of our synthesized LNMO NPs and NRs were determined to be 99.96% and 94.82%, respectively. That means almost fully ordered B-site occupancy in the LNMO NPs and only a small partial disorder (ASD content = 2.59%) at the B-site of the LNMO NRs. Therefore, the high quality of our LNMO samples was achieved. Their structural, dielectric, magnetic, and optical properties were comprehensively investigated, and the structure-property relationship in the nanostructured LNMO is discussed. This study will provide insights into understanding the structure-property relationship in the nanostructured LNMO, revealing the fundamental physical mechanisms behind them.

## 2. Materials and Methods

### 2.1. Sample Synthesis

In this work, both LNMO NPs and NRs were synthesized via the hydrothermal method. La(NO_3_)_3_·6H_2_O, Ni(NO_3_)_2_·6H_2_O, and MnCl_2_·4H_2_O were used as the starting materials, which were taken in a molar ratio of 2:1:1 and dissolved within 5 M NaOH solution under continued stirring at room temperature (RT). The mixed solution was transferred into a Teflon-lined hydrothermal vessel, which was placed in an oven heated at 180 °C for 24 h. The resultant powders obtained after cooling were washed with deionized water and filtrated, and then dried in air at 80 °C for 12 h. The brown powders were annealed at 1000 °C for 4 h. To remove the small amounts of NiO and La_2_O_3_ impurities, the as-synthesized LNMO powders were washed with dilute nitric acid and then dried to obtain the products with pure phase structure. For the synthesis of the LNMO NRs, the concentration of NaOH was 20 M, and the hydrothermal process was performed at 180 °C for 48 h, and the annealing process was carried out at 900 °C for 4 h.

In order to measure the dielectric properties of the synthesized NPs (NRs), the LNMO NPs (NRs) were pressed into pellets with a diameter of 10 mm under a pressure of 12 MPa, which were annealed at 650 °C for 2 h in atmospheric air. The densities of the LNMO ceramics prepared from the LNMO NPs and NRs, were measured to be 3.55 g/cm^3^ and 3.74 g/cm^3^, respectively. The two sides of the ceramic pellets were coated with silver paste and then cured at 540 °C for 2 h to form the electrodes for dielectric measurements.

### 2.2. Sample Characterization

At room temperature, powder X-ray diffraction patterns of the as-synthesized LNMO NPs and NRs were recorded by using a Bruker D8 diffractometer (Bruker D8 Advance, Bruker AXS GmbH, Karlsruhe, Germany) under Cu Kα irradiation at 40 kV and 30 mA. The XRD patterns were collected from 2θ = 15° to 85° and a typical scan rate was 0.02° per second. The collected XRD data were analyzed by Rietveld refinement using GSAS software (http://doc.wendoc.comnl.gov/public/gsas/, accessed on 25 January 2007, GSAS-II, Los Alamos National Laboratory (Los Alamos, NM, USA)), which allowed the refinements of structural parameters. The surface morphology of the as-synthesized LNMO samples was revealed by scanning electron microscope (SEM, S-3400N Ⅱ, Hitachi, Tokyo, Japan, operated at 25 kV) equipped with an energy dispersive X-ray spectrometer (EDS, EX-250 spectroscopy, HORIBA Corporation, Kyoto, Japan) analyzer. X-ray photoelectron spectroscopy (XPS) spectra recorded in the XPS-PHI 5000 Versa Probe (ULVAC-PHI, Kanagawa, Japan) under Al Kα irradiation, were used to determine the chemical oxide states of the elements in the LNMO samples. The XPS resolution is 0.025 eV. The XPS data were processed using CasaXPS software and calibrated with a C 1s peak with binding energy (BE) of 284.60 eV. Dielectric measurements of the LNMO ceramic samples were performed by using a computer-controlled Agilent 4192 A impedance analyzer (Agilent technologies, Santa Clara, CA, USA) in the frequency range of 10^2^–10^6^ Hz and the temperature was controlled from 180 K to 300 K by an automated temperature controller (DMS-2000, TH2828s, Partulab Technology, Wuhan, China).

Magnetic data of the as-synthesized samples were collected by a quantum design vibrating sample magnetometer (VSM) (model: MPMS-XL-5, Quantum Design, San Diego, CA, USA) under ZFC and FC modes with applied magnetic fields of 100 Oe and 1000 Oe, respectively, where the temperature was in the range of 5–300 K. During the ZFC mode measurements the samples were cooled in absence of a magnetic field from room temperature down to 5 K. At 5 K, a steady magnetic field of 100 Oe was applied, and the temperature was increased from 5 K to room temperature with a slow rate of 0.2 K/min. The data were acquired during the warming process. The samples were further cooled down to 5 K from room temperature under a magnetic field of 100 Oe to record the *M*(T) magnetization in FC mode. The above processes were repeated as the external magnetic field was changed to 1000 Oe for the LNMO NPs and LNMO NRs. Magnetic field (*H*) dependent magnetization (*M*) (*M*-*H* hysteresis loop) was measured with *H* up to 5 T at 5 K and 300 K, respectively. The absorption spectra of the LNMO samples were recorded by using a UV-Vis spectrophotometer (UV-3600, Shimadzu Co., Nakagyo-ku, Kyoto, Japan) at RT with the wavelength in the range of 200 nm to 800 nm, from which the optical bandgaps were determined by using the Tauc equation.

## 3. Results and Discussion

### 3.1. Structural Characterization

**Figure 1:**
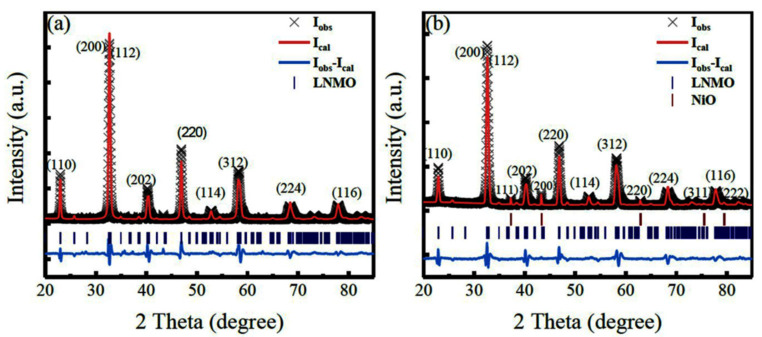
XRD patterns of the as-synthesized LNMO samples. (**a**) LNMO NPs and (**b**) LNMO NRs.

Figure 1a,b presents the RT XRD patterns of the LNMO NPs and NRs, respectively. They could be well indexed on monoclinic (*P*2_1_/*n*) cells. In addition, the XRD data were also refined by GSAS software based on the *P*2_1_/*n* model of the DP structure, as shown in Figure 1. A good match between the refined XRD patterns and the experimental ones is obtained, as confirmed by the fitting parameters (R_p_ = 8.93% and R_wp_ = 12.04% for LNMO NPs, and R_p_ = 8.54%, R_wp_ = 11.72% for LNMO NRs). However, a small amount of NiO impurity (weight percent about 5.80%) was observed in the LNMO NRs, whereas it was completely removed in LNMO NPs by acetic acid washing. The relevant refined structural parameters (e.g., unit cell parameters (*a*, *b*, and *c*, and cell volume (*V*), and the selected bond distances and angles), as well as the fitting parameters (Rp and Rwp), are presented in Table 1. For LNMO NPs, the lattice parameters are *a* = 5.5060(6) Å, *b* = 5.4482(5) Å, *c* = 7.7569(9) Å, *β* = 89.91(3)°; and *a* = 5.5087(6) Å, *b* = 5.4455(3) Å, *c* = 7.7736(8) Å, *β* = 89.91° for LNMO NRs. It is found that the refined unit cell parameters (*a*, *b*, and *c*) of the as-synthesized LNMO samples are smaller than that of the bulk sample [32], but the difference between the NPs and the NRs samples is not significant. There was a structural distortion in the as-synthesized LNMO samples, as revealed by the deviation of the <Ni-O-Mn> bond angle (~160.6°) from 180° as well as the deviation of *β* angle (~89.9°) from 90°. To describe the structural distortion produced in the LNMO samples, Goldschmidt structural tolerance factor (*t*) is utilized, which is expressed as [6]:

(1) $$t=\frac{r_{\mathrm{La}}+r_{O}}{\sqrt{2}\left(\frac{<r_{\mathrm{Ni}}>+\langle r_{\mathrm{Mn}}\rangle }{2}+r_{O}\right)}$$

where *r*_La_ and $r_{O}$ are the ionic radii of La^3+^ and O^2−^ ions, and $<r_{\mathrm{Ni}}>\mathrm{and}\langle r_{\mathrm{Mn}}\rangle$ are the average ionic radii of Ni and Mn ions. Considering the different charge states of Ni and Mn in the LNMO NPs, $\mathrm{the}\;\mathrm{value}\;\mathrm{of}\;<r_{\mathrm{Ni}}>$ is calculated to be 0.69 × 49% + 0.60 × 51% = 0.64 Å; and $<r_{\mathrm{Mn}}>$ is calculated as 0.645 × 42% + 0.53 × 58% = 0.58 Å. Thus, the *t* value for the LNMO NPs is calculated to be 0.901 based on the following Shannon’s ionic radii: $r_{{\mathrm{La}}^{3+}}$ = 1.16 Å (eight coordination number, C.N. = 8), $r_{{\mathrm{Ni}}^{2+}}$ = 0.69 Å (C.N. = 6),$\;r_{{\mathrm{Ni}}^{3+}}$ = 0.60 Å (C.N. = 6, high spin),$\;r_{{\mathrm{Mn}}^{3+}}$ = 0.645 Å (C.N. = 6, high spin),$\;r_{{\mathrm{Mn}}^{4+}}$ = 0.53 Å (C.N. = 6), and $r_{O^{2-}}$ = 1.40 Å (C.N. = 6) [53]. Similarly, the *t* value for the LNMO NRs is calculated to be 0.905. If the *t* value approaches 1.0, cubic perovskite is preferred to be formed at RT. While for 0.96 < *t* < 1.0, at RT a rhombohedral perovskite prefers to be formed, whereas it can be orthorhombic or monoclinic perovskite for lower *t* values [54]. The calculated *t* value for LNMO NPs is 0.901 (0.905 for LNMO NRs), smaller than 0.96, which means a structural distortion was generated from cubic perovskite to a monoclinic perovskite. That is reflected in the deviation of *β* angle from 90° and the <Ni-O-Mn> bond angle (~160.6°) from 180°. 

**Figure 2:**
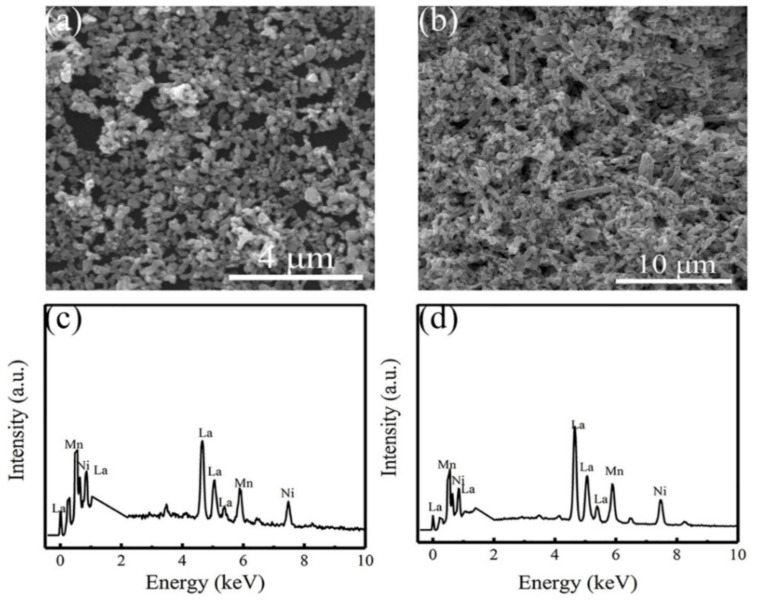
SEM images of the as-synthesized LNMO samples. (**a**) LNMO NPs and (**b**) LNMO NRs. EDS spectra of the as-synthesized (**c**) LNMO NPs and (**d**) LNMO NRs.

Figure 2a,b displays the typical SEM images of the as-synthesized LNMO NPs and NRs, respectively. It is observed that the LNMO NPs (Figure 2a) are uniformly distributed and have similar particle sizes, roughly ~260 ± 60 nm. The SEM image of the LNMO NRs (Figure 2b) reveals the presence of NRs in the field of view, which are non-uniformly distributed and composed of some NPs. That was mainly caused by high temperature annealing, leading to the destruction of the NR morphology. From the SEM image, the diameters of the LNMO NRs were estimated to be 430 ± 120 nm and the length about 2.05 ± 0.65 μm. Details are described in Figure S1. Actually, the LNMO NRs are assembled from NPs. The EDS spectra of the as-synthesized LNMO of NPs and NRs collected in a mapping mode are shown in Figure 2c,d, respectively, which reveal the signals of the constituent elements. The quantitative EDS data determined the cationic atomic ratios of La, Ni, and Mn elements for the LNMO NPs equal to 1.90(7):1.00:1.20(3), and 1.90(5):1.00: 1.08(2) for the LNMO NRs. They approach the stoichiometric value of 2:1:1.

**Figure 3:**
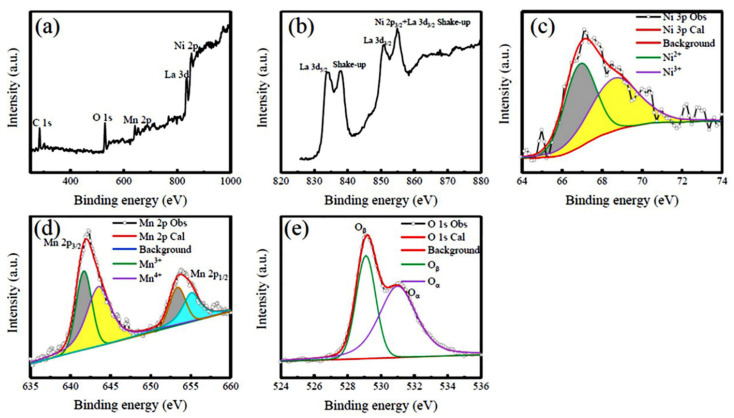
XPS spectra and peaking fitting of the LNMO NPs. (**a**) Survey scan XPS spectrum, (**b**) La 3d and Ni 2p, (**c**) Ni 3p, (**d**) Mn 2p, and (**e**) O1s XPS spectra.

**Figure 4:**
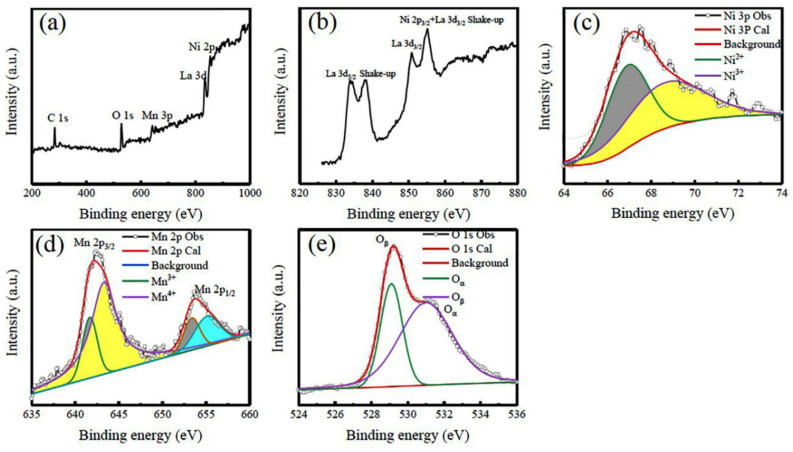
XPS spectra and peaking fitting of the LNMO NRs. (**a**) Survey scan XPS spectrum, (**b**) La 3d and Ni 2p, (**c**) Ni 3p, (**d**) Mn 2p, and (**e**) O1s XPS spectra.

To obtain more insights into the chemical structures of the LNMO NPs and NRs, peak fitting analyses of the La 3d, Ni 2p, Mn 2p and O 1s XPS spectra have been obtained and the fitting results are demonstrated in Figure 3 and Figure 4, respectively. Figure 3a displays the survey scanning XPS spectrum of the LNMO NPs, which verifies all the expected elements. In addition, the C 1s XPS line located 284.60 eV was also observed, which resulted from the adhesive carbon tape used during the XPS measurements, and was also used to calibrate the XPS data. Local La 3d and Ni 2p XPS spectra are shown in Figure 3b, which exhibit complex features because of the spin-orbit coupling and multiplet splitting. It is noticed that the La 3d_5/2_ XPS spectrum is split into two peaks located at 833.7 eV and 837.2 eV, respectively, indicating the presence of La in the +3 oxidation state in the LNMO NPs [55]. Around 850 eV, the Ni 2p_3/2_ peak and La 3d_3/2_ satellite coincide with each other [56]. Therefore, it was impossible to precisely distinguish from the Ni^2+^ and Ni^3+^ states in the LNMO NPs. However, the Ni 3p XPS spectrum is relatively easier to be examined as it is not interfered with by any La or other signals.

Thus, the Ni 3p XPS spectrum was measured, as shown in Figure 3c. It was deconvoluted into two peaks located at 66.90 eV and 68.60 eV, respectively, corresponding to the measured, as shown in Figure 3c. It was deconvoluted into two peaks located at 66.90 eV and 68.60 eV, respectively, corresponding to the Ni^2+^ and Ni^3+^ ions, respectively [57]. That means the existence of dual chemical states of Ni ions, and the molar ratio of [Ni^2+^] to [Ni^3+^] is calculated to be 49%:51%. As shown in Figure 3d, the Mn 2p XPS spectrum exhibits the Mn 2p_3/2_ and Mn 2p_1/2_ two peaks, which are located at 641.96 eV and 653.60 eV, respectively, due to the spin-orbit coupling. The Mn 2p_3/2_ XPS peak can be deconvoluted into two components located at 641.67 eV and 643.51 eV, respectively, corresponding to the Mn^+3^ ions, whereas the Mn 2p_1/2_ XPS peak is also deconvoluted into two components that are located 653.35 eV and 655.10 eV, respectively, corresponding to the Mn^4+^ ions [58]. The molar ratio of [Mn^3+^] to [Mn^4+^] is estimated to be 42%:58%. The O1s XPS spectrum (Figure 3e) displays an intense peak at a BE position of 529.12 eV and a distinct shoulder at the higher BE side. The O 1s spectra are deconvoluted into two peaks with BE positions at 529.10 eV and 531.12 eV, respectively, which indicate the existence of two different oxygen species in the LNMO NPs and NRs [59,60,61,62,63]. According to the previous reports, the peak with the lower BE position at 529.10 eV is assigned to the lattice oxygen (O^2-^) (denoted as O_β_) [59,60,61,62,63], while the assignment of the other peak with a higher BE position at 531.12 eV is controversial. It is possibly due to oxygen being weakly bound on the surface [60], surface hydroxyls, or oxygen bound to basic elements (e.g., La_2_O_3_) [64]. Tejuca et al. [63] assigned this peak to adsorbed oxygen species normally denoted as α-oxygen (O_α_). Based on a series of O1s XPS data of metal oxides, hydroxides and perovskite oxides, Dupin et al. [65] assigned the O 1s XPS peaks with higher BE values in the range of 531–532 eV to the “O^−^” ions. These ionizations of oxygen species are associated with sites where the coordination numbers of oxygen ions are smaller than that in regular sites, exhibiting a higher covalence of the M-O bonds and allowing for the compensation of deficiencies in the subsurface of transition metal oxides. Due to a higher covalence of the M-O bonds these low coordinated oxygen ions have a lower electron density than the classical “O^2−^ “ions. Since the present LNMO NPs and NRs were synthesized by a hydrothermal process and then followed annealing at 1000 °C and 900 °C for 4 h, respectively; therefore, the presence of hydroxyls at the surface of the LNMO samples is negligible. It is noticed that the concentration of the adsorbed oxygen (“O^−^” ions) is higher, which implies more O^−^ ions in this kind of complex oxide at the surface. 

The above XPS results reveal that there are dual chemical states of Ni and Mn ions, which differ from the standard bulk LNMO with an ideal ordered rock salt structure where the Mn and Ni ions appear as +4 and +2 oxidation states [66], respectively. Based on the data of Ni 3p and Mn 2p XPS spectra, the actual chemical oxidation states of Ni and Mn ions in the LNMO NPs are calculated to be +2.51 and +3.58, respectively. By the same method, the XPS spectra of the LNMO NRs (shown in Figure 4) are also analyzed, which are similar to the case of LNMO NPs. The molar ratio of [Ni^2+^] to [Ni^3+^] was calculated to be 48%:52%, and 22%:78% for the [Mn^3+^]/[Mn^4+^] molar ratio. Thus, the true chemical oxidation states of Ni and Mn ions are +2.52 and +3.78, respectively. 

### 3.2. Dielectric Properties 

**Figure 5:**
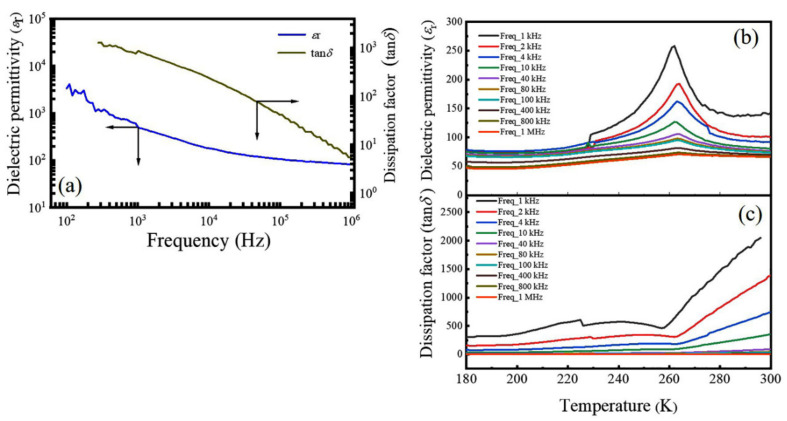
(**a**) Room temperature frequency dependence of the dielectric permittivity (*ε*_r_) and dissipation factor (tan*δ*) of the LNMO ceramics prepared from NPs. (**b**,**c**) Corresponding temperature dependence of the dielectric permittivity and dissipation factor of the LNMO ceramics measured at different frequencies.

Figure 5a demonstrates the frequency dependence of the dielectric permittivity (*ε*_r_) and dissipation factor (tan*δ*) of the LNMO ceramics (prepared from NPs) at RT. It is observed that the *ε*_r_ value decreases with increasing frequency. In the low frequency region, a very high value of *ε*_r_ is observed, whereas in the high frequency region it decreases fast to a very small value and becomes approximately independent of the frequency. In general, electronic, ionic, dipolar and interfacial polarizations can contribute to the dielectric permittivity of any material. At low frequencies, the dipolar and interfacial polarizations are effective to the dielectric permittivity. In contrast, at higher frequency the electronic polarization is effective, and the dipolar contribution becomes less insignificant. In other words, the higher value of *ε*_r_ at a lower frequency can be ascribed to the presence of different types of polarization, particularly the space charge polarization. Conversely, a nearly constant value of *ε*_r_ observed in higher frequency region is due to the absence of space-charge polarization [67]. The space charge polarization may be resulted from the creation of oxygen vacancies ($V_{0}^{‥}$) in the LNMO ceramic samples sintered at high temperatures. The XPS spectra reveal the dual chemical valence states of Ni (Ni^2+^ and Ni^3+^) and Mn (Mn^3+^ and Mn^4+^) in the present LNMO samples; therefore, the valence fluctuations in V ions and Fe ions also enhance the space charge polarization. The electronic hopping between Ni^2+^ and Ni^3+^ ions as well as Mn^3+^ and Mn^4+^ at the octahedral site of B′ and B′′ cations along the field direction also contribute to the high value of *ε*_r_. At high frequency, the charge carriers (like electrons) are unable to catch up with the applied field which results in the decrease of dielectric polarization and hence the reduction of *ε*_r_ value. Such dielectric behavior with a strong frequency dispersion observed in the LNMO ceramics can be ascribed to the space charge polarization and the $V_{0}^{‥}$ induced dielectric relaxation.

Figure 5b presents temperature dependence of the value of *ε*_r_ of the LNMO ceramics at different frequencies. As shown in Figure 5b, the *ε*_r_ value first increased with temperature and reached a maximum value around 262 K (*T*_max_ = 262 K), and then decreased with further increasing temperature. It is also noticed that the maximum value of *ε*_r_ collapses with increasing frequency. This dielectric anomaly suggests the occurrence of ferroelectric—paraelectric phase transition at 262 K. In addition, it is also observed that the value of *T*_max_ is almost independent of the frequency, indicating that the LNMO ceramics do not behave as relaxor-type ferroelectrics. It is well known that in normal relaxor dielectrics, the peak temperature (*T*_m_) at which the value of *ε*_r_ reaches a maximum is frequency dependent and shifts to higher temperatures with increasing the measured frequency. In addition, the maximum value of *ε*_r_ also decreases simultaneously [68]. Figure 5c exhibits the tan*δ* variation with respect to the temperature measured at different frequencies, where the tan*δ* value first increased with the frequency, then decreased to a minimum value around 262 K. However, above the temperature of 262 K, the tan*δ* increased linearly again. This phenomenon is attributed to the thermally activated carriers and defects in the LNMO ceramics. They result in an increase of the conductivity of the ceramic sample with the temperature, leading to a much larger value of tan*δ* at the high temperature region [69].

To confirm the ferroelectric—paraelectric phase transitions taking place in the LNMO ceramics prepared from the NPs and NRs, temperature dependent *ε*_r_ and its reciprocal (1000/*ε*_r_) is shown in Figure S2. In Figure S2a a pronounced anomaly of *ε*_r_ is observed at the ferroelectric Curie temperature *T*_C_ = 262 K with a peak value about five times the magnitude of the room-temperature value. Such dielectric response exhibits much smaller frequency dispersion (only 2 K in the frequency range of 1 kHz–1 MHz) as compared with the typical frequency dispersion of ~30 K in a normal relaxor ferroelectric Ba(Ti_0.70_Sn_0.30_)O_3_ [70]. In the corresponding plot of 1000/*ε*_r_—*T* curve, the Curie–Weiss law is followed in the temperature range of 230–280 K, but this temperature range is separated by a Curie–Weiss temperature *T*_0_, equal to the Curie temperature *T*_C_. Therefore, the ferroelectric—paraelectric phase transition occurs at *T*_c_ = 262 K, which belongs to a second-order transition. A similar case was previously reported in a LiTaO_3_ single crystal [71]. In the LNMO ceramics prepared from NRs, the ferroelectric—paraelectric phase transition appears at *T*_c_ = 260 K, belonging to a second-order phase transition, as shown in Figure S2c,d. 

To explore the nature of the dielectric behavior observed in the LNMO ceramics, a modified Curie–Weiss law is used [72],

(2) $$\frac{1}{\varepsilon_{r}}-\frac{1}{\varepsilon_{m}}=\frac{{\left(T-T_{m}\right)}^{\gamma }}{C}$$

where *ε*_r_ is the dielectric permittivity, *ε*_m_ is the maximum value of dielectric permittivity at *T*_m_, *C* is the Curie–Weiss constant, and *γ* is the diffusion phase transition coefficient, which varies from 1.0 (for normal-type ferroelectrics) [73] to 2.0 (for ideal relaxor-type ferroelectrics) [74]. Figure S3 shows the plots of ln ($\frac{1}{\varepsilon_{r}}-\frac{1}{\varepsilon_{m}})$ as a function of ln (*T* − *T*_m_) for the LNMO ceramics (prepared from NPs) measured at different frequencies, which exhibit a linear relationship, and the *γ* values are determined from the slopes of the fitting curves. The obtained *γ* values are 1.47 (@ 4 kHz), 1.23 (@ 10 kHz), 1.21 (@ 80 kHz), and 1.20 (@ 100 kHz), respectively, which are larger than 1.0 but smaller than 2.0, indicating the LNMO ceramic samples are neither normal ferroelectrics nor an ideal relaxor-type ferroelectric, but more like normal ferroelectrics. A similar conclusion is effective for the LNMO ceramics prepared from NRs, where the obtained *γ* values are in the range of 1.10–1.28 (see in Figure S4), closer to *γ* = 1.0.

**Figure 6:**
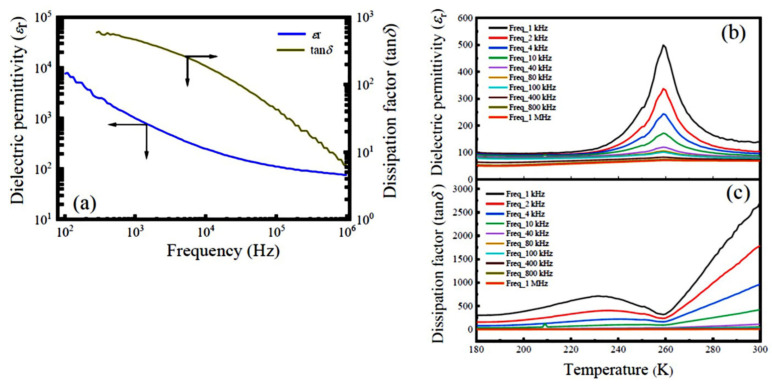
(**a**) Room temperature frequency dependence of the dielectric permittivity (*ε*_r_) and dissipation factor (tan*δ*) of the LNMO ceramics prepared from NRs. (**b**,**c**) Corresponding temperature dependence of the dielectric permittivity and dissipation factor of the LNMO ceramics measured at different frequencies.

Figure 6 represents the frequency dependence of the dielectric properties of the LNMO ceramics (prepared from LNMO NRs) measured at RT and the temperature dependent dielectric properties measured under a series of frequencies. As compared with the LNMO ceramics prepared from the NPs, the LNMO ceramics prepared from NRs exhibit higher *ε*_r_ and smaller tan*δ*, whereas their temperature dependence of the dielectric properties exhibits a similar dielectric temperature spectrum. The ferroelectric –paraelectric phase transition appeared around 260 K, and the second-order phase transition was identified in this sample.

### 3.3. Magnetic Properties 

**Figure 7:**
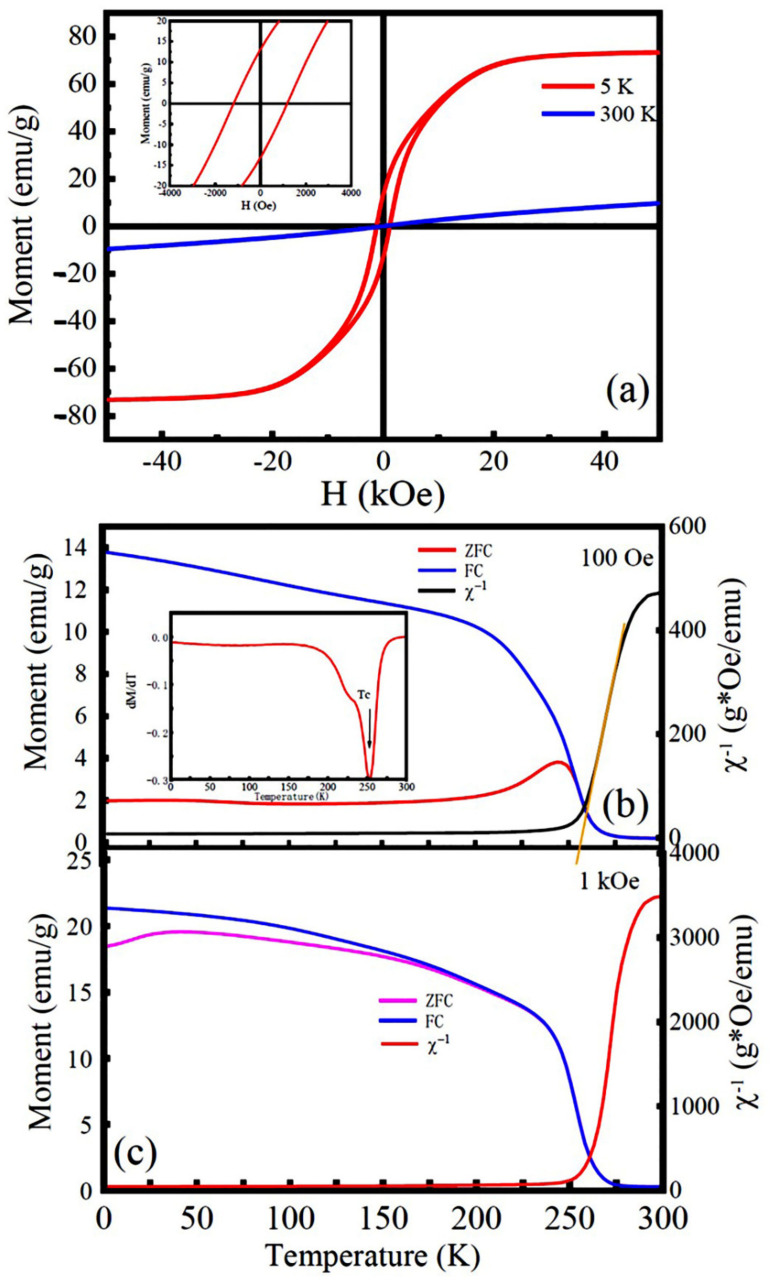
(**a**) M-H hysteresis loops of the LNMO NPs recorded at 5 K and 300 K. Inset is local enlarged *M*-*H* curve. (**b**,**c**) Temperature dependent dc magnetizations measured under ZFC and FC modes with applied magnetic fields of 100 Oe and 1000 Oe, respectively, and the temperature dependence of reciprocal ZFC magnetic susceptibilities, *χ*^−1^ measured at 100 Oe and 1000 Oe (right Y column), respectively. Inset in Figure b is d*M*_ZFC_/d*T* versus temperature to determine the magnetic Curie temperature (*T*_C_).

Figure 7a shows the field-dependent dc magnetizations of the LNMO NPs measured at different temperatures (e.g., 5 K and 300 K). At 300 K, the *M*-*H* curve is linear and exhibits paramagnetic characteristics. However, a typical ferromagnetic *M*-*H* hysteresis loop was observed at 5 K. From which the saturation magnetization (*M*_S_), remanent magnetization (*M*_r_), and coercive field (*H*_C_) were obtained, which were 71 emu/g (or 6.20 *μ*_B_/f.u.), 13.1 emu/g (or 1.14 *μ*_B_/f.u.), and 1141 Oe, respectively. Figure 7b, c shows the temperature dependence of dc magnetizations recorded under ZFC and FC modes with magnetic fields of 100 Oe and 1000 Oe, respectively. As shown as an inset in Figure 7b, the plot of d*M*_ZFC_/d*T* versus temperature leads to a local minimum, where the magnetic phase transition temperature (*T*_C_) was determined to be 250 K. As depicted in Figure 7b, the ZFC and FC dc magnetization curves diverge around the temperature, *T*_irr_ = 250 K, and larger bifurcation appears with further reducing the temperature. The ZFC magnetization curve also exhibits a small tip near 243 K, corresponding to the Neel temperature (*T*_N_). With increasing the applied magnetic field up to 1000 Oe, the *T*_irr_ temperature shifted to ~175 K from 250 K, as demonstrated in Figure 7c. It is also noticed that the bifurcations between the *M*_ZFC_ and *M*_FC_ curves are suppressed, indicating the presence of spin-glass ordering in the LNMO NPs [35].

**Figure 8:**
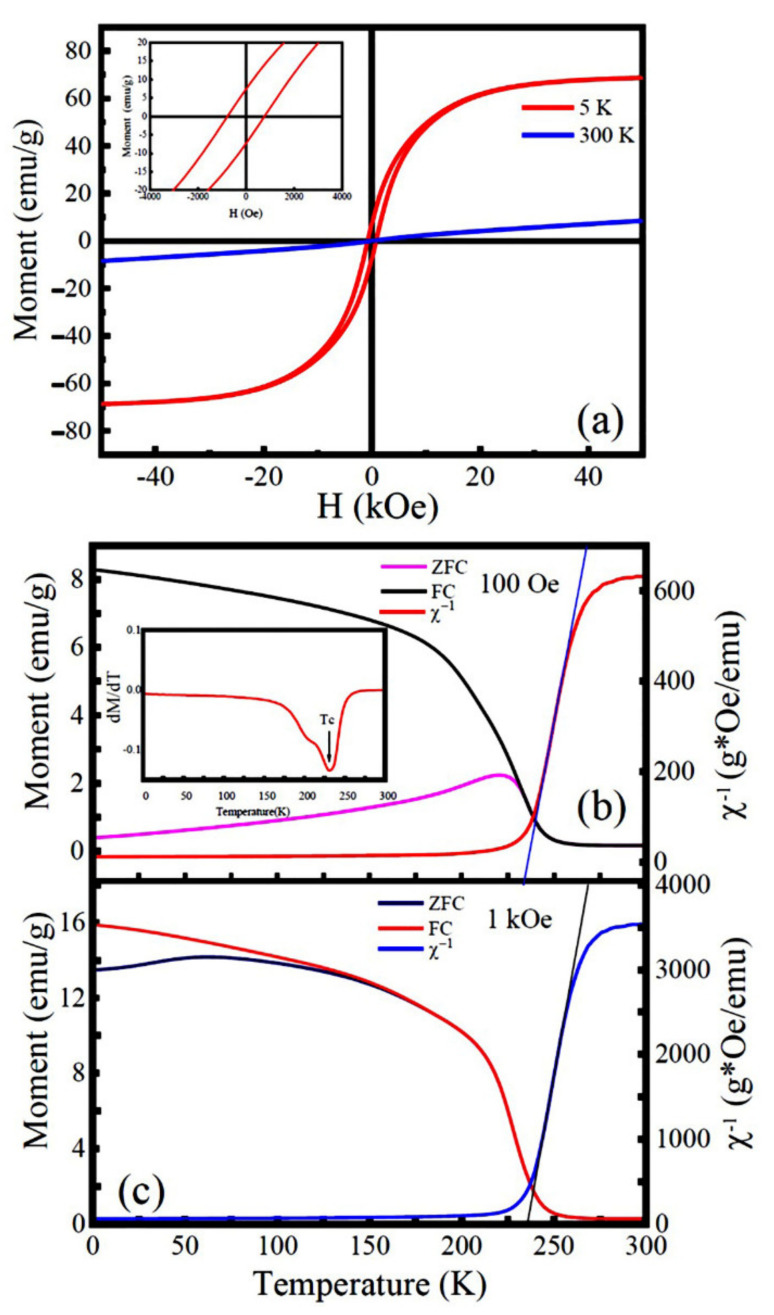
(**a**) *M*-*H* hysteresis loops of the LNMO NRs recorded at 5 K and 300 K. Inset is local enlarged M-H curve. (**b**,**c**) Temperature dependent dc magnetizations measured under ZFC and FC modes with applied magnetic fields of 100 Oe and 1000 Oe, respectively, and the temperature dependence of reciprocal ZFC magnetic susceptibilities, *χ*^−1^ measured at 100 Oe and 1000 Oe (right Y column), respectively. Inset in (**b**) is d*M*_ZFC_/d*T* versus temperature to determine the magnetic Curie temperature (*T*_C_).

Figure 8 displays the *M*(*H*) data of the as-synthesized LNMO NRs measured at 5 K and 300 K, and the temperature dependent *M*(*T*), ZFC and FC dc magnetization under 100 Oe and 1000 Oe. The *T*_C_ and *T*_N_ were determined to be 232 K and 220 K, respectively. It is found that at 5 K the LNMO NRs exhibit a similar *M*-*H* hysteresis loop as compared with the LNMO NPs. Their *M*_S_, *M*_r_, and *H*_C_ values were determined to be 65 emu/g (5.68 *μ*_B_/f.u.), 7.36 emu/g (or 0.64 *μ*_B_/f.u.) and 763 Oe, respectively, which are smaller than that of the LNMO NPs. That is ascribed to the existence of ASD defects (ASD content = 2.59%) in the LNMO NRs. It is also noticed that the areas of the *M*-*H* hysteresis loop (or the hysteresis energy loss per cycle) of the LNMO NPs and NRs exhibit some differences. The LNMO NPs possess a larger hysteresis energy loss per cycle due to their larger *M* and *H*_C_ values, which enable them to be promising candidates to meet the requirement for a high maximum-energy [(*MH*)_max_] product (high-energy product permanent magnets) in the industry. As demonstrated above, the *M*_S_ values of LNMO samples synthesized by the hydrothermal method are higher than that of LNMO samples synthesized by other methods, such as bulk LNMO sample (*M*_s_ ≈ 5.0 *μ*_B_/f.u.) prepared by solid-phase reaction [3] and LNMO nanoparticle (*M*_s_ = 3.74 *μ*_B_/f.u.) synthesized via microwave sintering technique [75]. A more detailed comparison is given in Table S1.

The *M*_S_ value can be theoretical calculated by the following equation:

(3) $$M_{S}=\sum n_{i}S_{i}g_{i}\mu_{B}$$

where *n*_i_ is the number of spins of the *i*th species, *S*_i_ is the spin of the *i*th ion, and *g*_i_ is the Land spectroscopic splitting factor (for Mn *g*_i_ = 2.0 and for Ni *g*_i_ = 2.2), and *μ*_B_ is Bohr magneton. Combined with the XPS results, the molar ratio of [Ni^2+^] to [Ni^3+^] ions was 49%:51%, and [Mn^3+^]:[Mn^4+^] = 42%:58% for LNMO NPs. For the LNMO NRs, the molar ratio of [Ni^2+^] to [Ni^3+^] ions was 48%:52% and [Mn^3+^]:[Mn^4+^] = 22%:78%. The electronic configurations for Ni^2+^, Ni^3+^, Mn^3+^ and Mn^4+^ ions are given as Ni^2+^(3*d*^8^, $t_{2g}^{6}e_{g}^{2}$; S = 1.0), Ni^3+^(3*d*^7^, $t_{2g}^{6}e_{g}^{1}$; S = 1.5), Mn^3+^(3*d*^4^, $t_{2g}^{3}e_{g}^{1}$; S = 2.0), Mn^4+^(3*d*^3^, $t_{2g}^{3}e_{g}^{0}$; S = 1.5) under high-spin states. Thus, the *M*_S_ values for the LNMO nanoparticle and NRs are calculated by the following equations.

*M*_S_ = (0.49 × 1.0+0.51 × 1.5) × 2.2+(0.42 × 2.0+0.58 × 1.5) × 2 = 6.18 *μ*_B_ (for NPs) (4)

*M*_S_ = (0.48 × 1.0+0.52 × 1.5) × 2.2+(0.22 × 2.0+0.78 × 1.5) × 2 = 5.99 *μ*_B_ (for NRs) (5)

Thus, the *M*_S_ values of the LNMO nanoparticle and NRs are calculated to be 6.18 *μ*_B_/f.u. and 5.99 *μ*_B_/f.u., respectively. They match well with the experimental values (6.20 *μ*_B_/f.u. for NPs and 5.68 *μ*_B_/f.u. for NRs).

The nature of magnetic exchange interactions in the as-synthesized LNMO NPs was also investigated. Figure 7a,b shows the temperature dependence of reciprocal ZFC magnetic susceptibilities (*χ*^−1^) measured at 100 Oe and 1000 Oe, respectively. They were fitted by the Curie–Weiss law, which is expressed by Equation (6) [76]:

*χ*^−1^(*T*) = (*T* − *θ*_p_)/*C* (6)

where *C* is the Curie constant (determined by the slope of the linear curve), and θp represents the Curie–Weiss temperature determined by the intercept of the extending linear part of *χ*^−1^ − *T* plot on the temperature axis. It is noticed that the *χ*^−1^ − *T* plot exhibits a linear relationship in the high temperature region over *T*_C_, indicating the LNMO NPs are in the paramagnetic phase. The fitting parameters, such as Curie constants *C* and *θ*_p_ for the LNMO NPs and NRs are presented in Table 2. It is found that the *θ*_p_ values of the LNMO NPs and NRs are large and positive, which indicates the dominant FM interactions in the LNMO samples. The appearance of *θ*_p_ > *T*_C_ in the LNMO samples suggests the short-ranged ordered states existing in the slightly higher temperature than *T*_C_. That resulted from the magnetic frustration caused by the phase competition between FM and AFM phases. In comparison with the LNMO NPs, LNMO NRs have a lower *θ*_p_, indicating weak long-range FM exchange interactions in the LNMO NRs. That is ascribed to their relatively higher degree of structural disorder caused by the ASD defects (ASD content = 2.59%), which lead to enhanced AFM interactions via the Ni^2+^-O^2−^-Ni^2+^ and Mn^4+^-O^2−^-Mn^4+^ magnetic paths. Their effective magnetic moments (*μ*_eff_) in the paramagnetic phase can be calculated by the Equation (7) [26]:

(7) $$\mu_{\mathrm{eff}}=\sqrt{\frac{3k_{B}C}{N_{A}\mu_{B}^{2}}}=2.828\sqrt{C}$$

where *k*_B_ is the Boltzmann constant, *μ*_B_ is the Bohr magneton, and *N*_A_ is the Avogadro number. The *μ*_eff_ values for the LNMO NPs were 5.39(4) *μ*_B_/f.u. (@100 Oe) and 5.47(4) *μ*_B_/f.u. (@1000 Oe), while 5.97(2) *μ*_B_/f.u. (@100 Oe) and 4.45(1) *μ*_B_/f.u. (@1000 Oe) for the LNMO NRs. As compared with the *μ*_eff_, theoretical magnetic moment, *μ*_cal_ (per formula unit) for the La_2_${\mathrm{Ni}}_{x}^{2+}{\mathrm{Ni}}_{1-x}^{3+}{\mathrm{Mn}}_{y}^{3+}{\mathrm{Mn}}_{1-y}^{4+}$O_6_ is calculated by Equation (8) [77]:

(8) $$\mu_{\mathrm{cal}}=\sqrt{x\mu_{{\mathrm{Ni}}^{2+}}^{2}+\left(1-x\right)\mu_{{\mathrm{Ni}}^{3+}}^{2}+y\mu_{{\mathrm{Mn}}^{3+}}^{2}+\left(1-y\right)\mu_{{\mathrm{Mn}}^{4+}}^{2}}$$

where$\;\mu_{{\mathrm{Ni}}^{2+}}$= 2.83 *μ*_B_, $\mu_{Ni^{3+}}$= 3.87 *μ*_B_, $\mu_{{\mathrm{Mn}}^{3+}}$= 4.90 *μ*_B_, $\mu_{{\mathrm{Mn}}^{4+}}$= 3.87 *μ*_B_, are effective magnetic moments of the Ni^2+^, Ni^3+^, Mn^3+^ and Mn^4+^ ions in the high-spin states, respectively [78]. Combined with the XPS data, the *μ*_cal_ values of the LNMO NPs and LNMO NRs were calculated to be 5.51 *μ*_B_/f.u. and 5.35 *μ*_B_/f.u., respectively. It is found that the *μ*_cal_ value for the LNMO NRs, is slightly smaller than their *μ*_eff_ value under an external magnetic field of 100 Oe, which may be due to the existence of short-ranged FM interactions between Mn^3+^ and Mn^4+^ ions, and Ni^3+^ and Mn^3+^ ions as well as a small amount of NiO impurity in the LNMO NRs [79].

Based on the theoretical saturation magnetization (*M*_Scal_) and experimental saturation magnetization (*M*_Sexp_) data, the ASD contents in the LNMO NPs and NRs can be determined by the following equation [20]:

(9) $$\mathrm{ASD}=\frac{\left(1-\frac{M_{\mathrm{Sexp}}}{M_{\mathrm{Scal}}}\right)}{2}$$

It is found that the ASD contents in the LNMO NPs and NRs, were 0.02% and 2.59%, respectively. The B-site ordering degree (η) of the LNMO is related to its ASD content [80]:

η = 1 − 2 ∗ ASD(10)

where the ASD content can vary from ASD = 0.0% (or η = 100%), corresponding to a completely ordered double perovskite to ASD = 50.0% (or η = 0.0), corresponding to a fully random Ni-Mn site occupancy. Based on the ASD contents of the LNMO NPs and NRs obtained from Equation (9), the η values of the LNMO NPs and NRs were determined to be 99.96% and 94.82%, respectively. That means nearly fully ordered B-site occupancy in the LNMO NPs and a small partial disorder at the B-site of the LNMO NRs.

### 3.4. Optical Properties 

**Figure 9:**
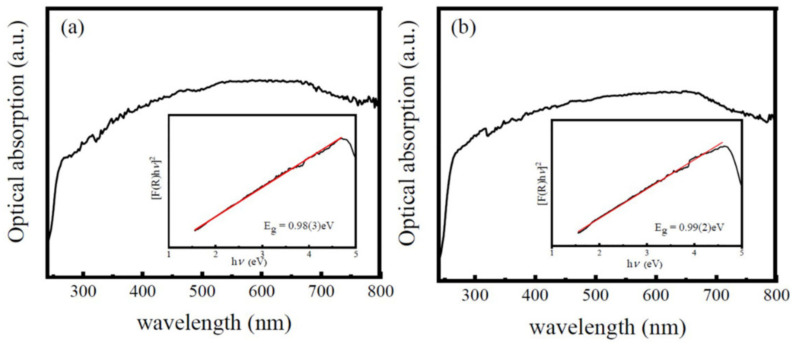
(Optical absorption spectra of (**a**) LNMO NPs and (**b**) NRs, and the insets are the corresponding Tauc plots of [*F*(*R*) × hν)]^2^ versus photon energy *hν*.

The absorption spectra of the LNMO NPs and NRs are depicted in Figure 9a,b, respectively. It is observed that both samples show sharp absorption peaks around 240 nm, indicating their large optical band gaps (*E*_g_). To determine the types of bandgap transition and the *E*_g_ values of the as-synthesized samples, the Tauc equation is utilized [81]:

(11) $$F\left(R\right)\times h\upsilon =B{\left(h\nu -E_{g}\right)}^{n}\;\;\;$$

where *F*(*R*) is the Kubelka–Munk function, expressed as *F*(*R*) *=* (1 − *R*)^2^/2*R*, and *R* is the reflectance. *hv* is photon energy, *B* is proportional constant, and *E*_g_ is the gap energy. The n values can change with respect to the transition types in a material, where n = 1/2 represents the direct allowed transition but n = 2 indicates an indirect and allowed transition. The Tauc plots of the [*F*(*R*) × *hv*]^2^ vs*. hv* curves are shown as insets in Figure 9. Their intersections of the tangent lines with the x-axis give out the *E*_g_ values of the LNMO NPs and NRs, which are 0.99(3) eV and 0.99(2) eV, respectively, indicating the semiconductor optical properties. The present *E*_g_ of the LNMO NPs is smaller than that reported previously for the LNMO NPs with *E*_g_ = 1.20 eV [36] and *E*_g_ = 1.08 eV [37]. In addition, the present *E*_g_ value of the LNMO NRs is also smaller than that reported for the LNMO NRs (*E*_g_ = 1.90 eV) synthesized by hydrothermal processes [46]. That may be possibly due to their different geometrical sizes and the different structural distortion of the DP crystal structure. It is reported that the *p-d* charge transfer from the O 2*p* orbital to Ni/Mn 3*d* orbitals contributes to the formation of direct optical band gaps in the LNMO NPs and NRs [46,82].

## 4. Conclusions

In summary, LNMO NPs and NRs were synthesized via a hydrothermal process, where only non-toxic aqueous inorganic solvents were utilized to modulate the microscopic morphology of the products without using any organic template agent. Their structural, dielectric, magnetic, and optical properties were studied. Rietveld structural refinements on the XRD data demonstrated that LNMO NPs and NRs crystallized in a monoclinic structure with a *P*2_1_/*n* space group. A structural distortion from cubic perovskite to monoclinic structure appeared in the LNMO samples. SEM images demonstrate that the LNMO NPs have a spherical morphology with an average particle size of 260 ± 60 nm and that the LNMO NRs have diameters of 430 ± 120 nm and lengths of about 2.05 ± 0.65 μm. The EDS data gave out the atomic ratios of La, Ni, and Mn elements for the LNMO NPs and NRs, close to the stoichiometric value of 2:1:1. XPS spectra confirmed the dual chemical valence states of Ni (Ni^2+^ and Ni^3+^) and Mn (Mn^3+^ and Mn^4+^) elements in the LNMO NPs and NRs, and the La element presented as a La^3+^ ion, and oxygen in the manner of lattice oxygen and the adsorbed oxygen. The LNMO ceramics (prepared from NPs and NRs) exhibit a dielectric behavior with a strong frequency dispersion, which is ascribed to the space charge polarization and the oxygen vacancy induced dielectric relaxation. The ferroelectric—paraelectric phase transition in the LNMO ceramics was shown to be of second-order phase transition. Both LNMO NPs and NRs exhibit ferromagnetic behavior at 5 K but a paramagnetic feature at 300 K. The magnetic Curie temperature and Neel temperature of the LNMO NPs were found to be 250 K and 243 K, respectively, and the corresponding temperatures for the LNMO NRs were 232 K and 220 K, respectively. The *M*_S_ values of the LNMO NPs and NRs at 5 K were measured to be 6.20 *μ*_B_/f.u. and 5.68 *μ*_B_/f.u., respectively. The larger *M*_S_ value of the LNMO NPs was ascribed to their nearly fully ordered B-sited occupancy. Theoretical magnetic moments $\left(\mu_{\mathrm{cal}}\right)$ of the LNMO NPs and LNMO NRs were calculated to be 5.51 *μ*_B_/f.u. and 5.35 *μ*_B_/f.u., respectively. The slightly higher effective magnetic moment (*μ*_eff_ = 5.97(2) *μ*_B_/f.u.) at 100 Oe for LNMO NRs can be attributed to the short-ranged ferromagnetic interactions between Mn^3+^ and Mn^4+^ ions, Ni^2+^ and Ni^3+^ ions, as well as the small amount of NiO impurity in the LNMO NRs. The large and positive Curie–Weiss temperatures (*θ*_p_) were determined in the LNMO samples, suggesting their dominant ferromagnetic interactions. The appearance of *θ*_p_ > *T*_C_ in the LNMO samples demonstrates the existence of short-range ordered states in the temperature slightly above *T*_C_. That is owed to the magnetic frustration caused by the phase competition between the FM and AFM phases. In addition, a higher *θ*_p_ was observed in the LNMO NPs as compared with the LNMO NRs, which implies stronger long-range ferromagnetic exchange interactions in the LNMO NPs due to being almost free of ASD defects. The UV–visible spectroscopic analyses unveiled the semiconducting nature of the nanostructured LNMO with an optical band gap of 0.99 eV, which originated from the *p*-*d* charge transfer from the O 2*p* orbital to Ni/Mn 3*d* orbitals. The present results indicate that the LNMO NPs and NRs have promising potential for the next generation of optoelectronic and spintronic device applications.

## Figures and Tables

**Table 1:** Structural parameters obtained from Rietveld refinements of the XRD data of the as-synthesized LNMO NPs and NRs.

Materials	Space Group	Unit Cell Parameters	Average Bond Length (Å) <Mn-O>	Average Bond Length (Å) <Ni-O>	Bond Angle (°) Ni-O_i_-Mn (*i* = 1–3) <Ni-O-Mn>	Structural Tolerance Factor (*t*)	Fitting Parameters
LNMO NPs	*P*2_1_/*n*	*a* = 5.5060(6) Å	1.9656(2)	1.9656(2)	158.217(4) 162.550(3) 161.192(4) 160.653(3)	0.901	R_p_ = 8.93% R_wp_=12.0%
		*b* = 5.4482(5) Å					
		*c* = 7.7569(9)Å					
		*β* = 89.91(3)°					
		*V* = 232.691(5) Å^3^					
LNMO NRs		*a* = 5.5087(6) Å	1.9670(2)	1.9670(2)	158.253(3) 162.533(2) 161.160(3) 160.648(3)	0.905	R_p_ = 8.54% R_wp_=11.72%
		*b* = 5.4455(3) Å					
		*c* = 7.7736(8) Å					
		*β =* 89.85(2) °*V =* 232.193(5) Å^3^					

**Table 2:** Magnetic data of the as-synthesized LNMO NPs and NRs measured at 5 K and their Curie–Weiss fitting parameters.

Material	*T*_irr_ (K)	*T*_c_ (K)	*θ*_p_ (K)	*C* (emu⋅K/mol)	*μ*_eff_ (*μ*_B_)	*μ*_cal_ (*μ*_B_)	*M*_r_ (emu/g)	*H*_C_ (Oe)	*M*_Sexp_ (*μ*_B_/f.u.)	*M*_Scal_ (*μ*_B_/f.u.)	ASD Content
LNMO NPs	@ 100 Oe					5.51	13.1	1141	6.20	6.18	0.02%
	258.4	252.0	257.0	3.64(5)	5.39(4)						
	@ 1000 Oe										
	239.5	253.0	262.0	3.75(5)	5.47(4)						
LNMO NWs	@ 100 Oe					5.35	7.36	763	5.68	5.99	2.59%
	234.5	232.0	235.8	4.43(5)	5.95(4)						
	@ 1000 Oe										
	185.5	227.0	235.6	2.48(1)	4.45(1)						

*T*_irr_: the temperature at which the ZFC and FC dc magnetization curves diverge; *T*_C_: Curie temperature; *θ*_p_: Curie–Weiss temperature; *C*: Curie–Weiss constant; *μ*_eff_: effective magnetic moment; *μ*_cal_: theoretical magnetic moment; *M*_r_: remanent magnetization; *H*_C_: coercive field; *M*_Sexp_: experimental saturation magnetization; *M*_Scal_: theoretical saturation magnetization; and ASD: anti-site defect content.

## Data Availability

Corresponding author. The data are not publicly available due to privacy.

## Supplementary Materials

The following supporting information can be downloaded at: https://www.mdpi.com/article/10.3390/nano12060979/s1, Figure S1: (a) Histogram of LNMO NPs size distribution. (b) and (c) Histograms of the distributions of the length and diameter of the LNMO NRs; Figure S2: Temperature dependent dielectric permittivity (*ε*_r_) and its reciprocal (1000/*ε*_r_) of the LNMO ceramics prepared from (a,b) NPs and (c,d) NRs; Figure S3: Plots of $\ln\left(\frac{1}{\varepsilon_{r}}-\frac{1}{\varepsilon_{m}}\right)\;$as a function of $\ln\left(T-T_{m}\right)$ for the LNMO ceramics prepared from NPs measured at (a) 4 kHz, (b) 10 kHz, (c) 80 kHz, and (d) 100 kHz; Figure S4: Plots of $\ln\left(\frac{1}{\varepsilon_{r}}-\frac{1}{\varepsilon_{m}}\right)\;$as a function of $\ln\left(T-T_{m}\right)$ for the LNMO ceramics prepared from NRs measured at (a) 4 kHz, (b) 10 kHz, (c) 80 kHz, and (d) 100 kHz; Table S1: Comparison between the magnetic data and Curie-Weiss fitting parameters of the as-synthesized LNMO samples (nanoparticles (NPs), nanorods (NRs), thin films, and bulk) and that reported in the recent literatures. References [83,84] are cited in Supplementary Materials.

## References

[1] Retuerto M., Muñoz Á., Martínez-Lope M.J., Alonso J.A., Mompeán F.J., Fernández-Díaz M.T., Sánchez-Benítez J. (2015). Magnetic Interactions in the Double Perovskites R_2_NiMnO_6_ (R = Tb, Ho, Er, Tm) Investigated by Neutron Diffraction. Inorg. Chem..

[2] Kakarla D.C., Jyothinagaram K.M., Das A.K., Adyam V. (2014). Dielectric and Magnetodielectric Properties of R_2_NiMnO_6_ (R = Nd, Eu, Gd, Dy, and Y). J. Am. Ceram. Soc..

[3] Rogado N.S., Li J., Sleight A.W., Subramanian M.A. (2005). Magnetocapacitance and Magnetoresistance near Room Temperature in a Ferromagnetic Semiconductor: La_2_NiMnO_6_. Adv. Mater..

[4] Nasir M., Kumar S., Patra N., Bhattacharya D., Jha S.N., Basaula D.R., Bhatt S., Khan M., Liu S.W., Biring S. (2019). Role of Antisite Disorder, Rare-Earth Size, and Superexchange Angle on Band Gap, Curie Temperature, and Magnetization of R_2_NiMnO_6_ Double Perovskites. ACS Appl. Electron. Mater..

[5] Sobolev A.V., Glazkova I.S., Akulenko A.A., Sergueev I., Chumakov A.I., Yi W., Belik A.A., Presniakov I.A. (2019). Ni-61 Nuclear Forward Scattering Study of Magnetic Hyperfine Interactions in Double Perovskites A_2_NiMnO_6_ (A = Sc, in, Tl). J. Phys. Chem. C.

[6] Anderson M.T., Greenwood K.B., Taylor G.A., Poeppelmeier K.R. (1993). B-Cation Arrangements in Double Perov skites. Prog. Solid State Chem..

[7] Nasir M., Khan M., Kumar S., Bhatt S., Patra N., Bhattacharya D., Jha S.N., Biring S., Sen S. (2019). The Effect of High Temperature Annealing on the Antisite Defects in Ferromagnetic La_2_NiMnO_6_ Double Perovskite. J. Magn. Magn. Mater..

[8] Dass R.I., Goodenough J.B. (2003). Multiple Magnetic Phases of La_2_CoMnO_6-δ_ (0 <~δ <~0.05). Phys. Rev. B.

[9] Choudhury D., Mandal P., Mathieu R., Hazarika A., Rajan S., Sundaresan A., Waghmare U.V., Knut R., Karis O., Nordblad P. (2012). Near-Room-Temperature Colossal Magnetodielectricity and Multiglass Properties in Partially Disordered La_2_NiMnO_6_. Phys. Rev. Lett..

[10] Wang T., Wu H.Y., Sun Y.B., Xing R., Xv B., Zhao J.J. (2020). Physical Properties of Sr-Doped Double Perovskite La_2_NiMnO_6_. J. Supercond. Nov. Magn..

[11] Chakraborty T., Nhalil H., Yadav R., Wagh A.A., Elizabeth S. (2017). Magnetocaloric Properties of R_2_NiMnO_6_ (R=Pr, Nd, Tb, Ho and Y) Double Perovskite Family. J. Magn. Magn. Mater..

[12] Matte D., de Lafontaine M., Ouellet A., Balli M., Fournier P. (2018). Tailoring the Magnetocaloric Effect in La_2_NiMnO_6_ Thin Films. Phys. Rev. Appl..

[13] Gauvin-Ndiaye C., Baker T.E., Karan P., Masse E., Balli M., Brahiti N., Eskandari M.A., Fournier P., Tremblay A.M.S., Nourafkan R. (2018). Electronic and Magnetic Properties of the Candidate Magnetocaloric-Material Double Perovskites La_2_NiCoO_6_, La_2_NiMnO_6_, and La_2_NiFeO_6_. Phys. Rev. B.

[14] Yi W., Princep A.J., Guo Y., Johnson R.D., Khalyavin D., Manuel P., Senyshyn A., Presniakav I.A., Sobolev A.V., Matsushita Y. (2015). Sc_2_NiMnO_6_: A Double-Perovskite with a Magnetodielectric Response Driven by Multiple Magnetic Orders. Inorg. Chem..

[15] Dass R.I., Yan J.Q., Goodenough J.B. (2003). Oxygen Stoichiometry, Ferromagnetism, and Transport Properties of La_2-x_NiMnO_6+δ_. Phys. Rev. B.

[16] Asai K., Sekizawa H., Iida S. (1979). Magnetization Measurements and ^55^Mn NMR Studies of LaNi_0.5_Mn_0.5_O_3_. J. Phys. Soc. Jpn..

[17] Blasse G. (1965). Ferromagnetic Interactions in Non-Metallic Perovskites. J. Phys. Chem. Solids..

[18] Kanamori J. (1959). Superexchange Interaction and Symmetry Properties of Electron Orbitals. J. Phys. Chem. Solids.

[19] Nasir M., Khan M., Bhatt S., Bera A.K., Furquan M., Kumar S., Yusuf S.M., Patra N., Bhattacharya D., Jha S.N. (2019). Influence of Cation Order and Valence States on Magnetic Ordering in La_2_Ni_1-X_Mn_1+X_O_6_. Phys. Status Solidi.

[20] Chandrasekhar K.D., Das A.K., Mitra C., Venimadhav A. (2012). The Extrinsic Origin of the Magnetodielectric Effect in the Double Perovskite La_2_NiMnO_6_. J. Phys.: Condens. Matter..

[21] Bernal-Salamanca M., Konstantinovic Z., Balcells L., Pannunzio-Miner E., Sandiumenge F., Lopez-Mir L., Bozzo B., Herrero-Martin J., Pomar A., Frontera C. (2019). Nonstoichiometry Driven Ferromagnetism in Double Perovskite La_2_Ni_1-X_Mn_1+X_O_6_ Insulating Thin Films. Cryst. Growth Des..

[22] Joly V.L.J., Joy P.A., Date S.K., Gopinath C.S. (2002). Two Ferromagnetic Phases with Different Spin States of Mn and Ni in LaMn_0.5_Ni_0.5_O_3_. Phys. Rev. B.

[23] Bull C.L., Gleeson D., Knight K.S. (2003). Determination of B-Site Ordering and Structural Transformations in the Mixed Transition Metal Perovskites La_2_NiCoO_6_ and La_2_NiMnO_6_. J. Phys. Condens. Matter..

[24] Iliev M.N., Gospodinov M.M., Singh M.P., Meen J., Truong K.D., Fournier P., Jandl S. (2009). Growth, Magnetic Properties, and Raman Scattering of La_2_NiMnO_6_ Single Crystals. J. Appl. Phys..

[25] Singh M.P., Truong K.D., Jandl S., Fournier P. (2009). Long-Range Ni/Mn Structural Order in Epitaxial DoublePerovskite La_2_NiMnO_6_ Thin Films. Phys. Rev. B.

[26] Pal S., Govinda S., Goyal M., Mukherjee S., Pal B., Saha R., Sundaresan A., Jana S., Karis O., Freeland J.W. (2018). Effect of Anti-Site Disorder on Magnetism in La_2_NiMnO_6_. Phys. Rev. B.

[27] Yuan X., Li Q., Hu J., Xu M. (2013). Unusual Dynamic Magnetic Behavior of Polycrystalline La_2_NiMnO_6_. Phys. B.

[28] Das H., Waghmare U.V., Saha-Dasgupta T., Sarma D.D. (2008). Electronic Structure, Phonons, and Dielectric Anomaly in Ferromagnetic Insulating Double Pervoskite La_2_NiMnO_6_. Phys. Rev. Lett..

[29] Zhao S., Shi L., Zhou S., Zhao J., Yang H., Guo Y. (2009). Size-Dependent Magnetic Properties and Raman Spectra of La_2_NiMnO_6_ Nanoparticles. J. Appl. Phys..

[30] Masud M.G., Ghosh A., Sannigrahi J., Chaudhuri B.K. (2012). Observation of Relaxor Ferroelectricity and Multiferroic Behaviour in Nanoparticles of the Ferromagnetic Semiconductor La_2_NiMnO_6_. J. Phys. Condens. Matter.

[31] Hossain A., Atique Ullah A.K.M., Sarathi Guin P., Roy S. (2020). An Overview of La_2_NiMnO_6_ Double Perovskites: Synthesis, Structure, Properties, and Applications. J. Sol-Gel Sci. Technol..

[32] Booth R.J., Fillman R., Whitaker H., Nag A., Tiwari R.M., Ramanujachary K.V., Gopalakrishnan J., Lofland S.E. (2009). An Investigation of Structural, Magnetic and Dielectric Properties of R_2_NiMnO_6_ (R = Rare Earth, Y). Mater. Res. Bull..

[33] Kumar P., Ghara S., Rajeswaran B., Muthu D.V.S., Sundaresan A., Sood A.K. (2014). Temperature Dependent Magnetic, Dielectric and Raman Studies of Partially Disordered La_2_NiMnO_6_. Solid State Commun..

[34] Islam S.A.U., Andrabi F.A., Mohmed F., Sultan K., Ikram M., Asokan K. (2020). Ba Doping Induced Modifications in the Structural, Morphological and Dielectric Properties of Double Perovskite La_2_NiMnO_6_ Ceramics. J. Solid State Chem..

[35] Nasir M., Khan M., Rini E.G., Agbo S.A., Sen S. (2021). Exploring the Role of Fe Substitution on Electronic, Structural, and Magnetic Properties of La_2_NiMnO_6_ Double Perovskites. Appl. Phys. A.

[36] Lan C., Zhao S., Xu T., Ma J., Hayase S., Ma T. (2016). Investigation on Structures, Band Gaps, and Electronic Structures of Lead Free La_2_NiMnO_6_ Double Perovskite Materials for Potential Application of Solar Cell. J. Alloys Compd..

[37] Sheikh M.S., Ghosh D., Dutta A., Bhattacharyya S., Sinha T.P. (2017). Lead Free Double Perovskite Oxides Ln_2_NiMnO_6_ (Ln = La, Eu, Dy, Lu), A New Promising Material for Photovoltaic Application. Mater. Sci. Eng. B.

[38] Biswal A.K., Ray J., Babu P.D., Siruguri V., Vishwakarma P.N. (2014). Dielectric Relaxations in La_2_NiMnO_6_ with Signatures of Griffiths Phase. J. Appl. Phys..

[39] Sayed F.N., Achary S.N., Jayakumar O.D., Deshpande S.K., Krishna P.S.R., Chatterjee S., Ayyub P., Tyagi A.K. (2011). Role of Annealing Conditions on the Ferromagnetic and Dielectric Properties of La_2_NiMnO_6_. J. Mater. Res..

[40] Gaikwad V.M., Yadav K.K., Lofland S.E., Ramanujachary K.V., Chakraverty S., Ganguli A.K., Jha M. (2019). New Low Temperature Process for Stabilization of Nanostructured La_2_NiMnO_6_ and Their Magnetic Properties. J. Magn. Magn. Mater..

[41] Wu Z.Y., Ma C.B., Tang X.G., Li R., Liu Q.X., Chen B.T. (2013). Double-Perovskite Magnetic La_2_NiMnO_6_ Nanoparticles for Adsorption of Bovine Serum Albumin Applications. Nanoscale Res. Lett..

[42] Mao Y.B., Parsons J., McCloy J.S. (2013). Magnetic properties of double perovskite La_2_BMnO_6_ (B = Ni or Co) nanoparticles. Nanoscale..

[43] Mao Y.B. (2012). Facile Molten-Salt Synthesis of Double Perovskite La_2_BMnO_6_ nanoparticles. RSC Adv..

[44] Yang D.X., Jiang R., Zhang Y.H., Zhang H., Lei S.L., Yang T., Hu X.S., Huang S., Ge J.Y., Su K.P. (2020). Effect of B-Site Ordering on the Magnetic Order in Multifunctional La_2_NiMnO_6_ Double Perovskite. Chin. Phys. Lett..

[45] De Azevedo Filho J.B., Souza R.F., Queiroz J.C.A., Costa T.H.C., Sena C.P.S., Fonseca S.G.C., da Silva A.O., Oliveira J.B.L. (2021). Theoretical and Experimental Investigation of the Structural and Magnetic Properties of La_2_NiMnO_6_. J. Magn. Magn. Mater..

[46] Gaikwad V.M., Yadav K.K., Sunaina Chakraverty S., Lofland S.E., Ramanujachary K.V., Nishanthi S.T., Ganguli A.K., Jha M. (2019). Design of Process for Stabilization of La_2_NiMnO_6_ Nanorods and Their Magnetic Properties. J. Magn. Magn. Mater..

[47] McGuire S.C., Koenigsmann C., Chou C.C., Tong X., Wong S.S. (2021). Lanthanum-based double perovskite nanoscale motifs as support media for the methanol oxidation reaction. Catal. Sci. Technol..

[48] Das H., Waghmare U.V., Saha-Dasgupta T., Sarma D.D. (2009). Theoretical Evidence and Chemical Origin of the Mag netism-Dependent Electrostructural Coupling in La_2_NiMnO_6_. Phys. Rev. B.

[49] Sheikh M.S., Sakhya A.P., Dutta A., Sinha T.P. (2019). Origin of Narrow Band Gap and Optical Anisotropy in Solar Cell Absorbers L_2_NiMnO_6_ (L La, Eu): A comparative DFT study. Comput. Mater. Sci..

[50] Pacheco J.M., Gueorguiev G.K., Martins J.L. (2002). First-Principles Study of the Possibility of Condensed Phases of En dohedral Silicon Cage Clusters. Phys. Rev. B.

[51] Oliveira M.I.A., Rivelino R., Mota F.D., Gueorguiev G.K. (2014). Optical Properties and Quasiparticle Band Gaps of Transition-Metal Atoms Encapsulated by Silicon Cages. J. Phys. Chem. C.

[52] Oliveira M.J.T., Medeiros P.V.C., Sousa J.R.F., Nogueira F., Gueorguiev G.K. (2014). Optical and Magnetic Excitations of Metal-Encapsulating Si Cages: A Systematic Study by Time-Dependent Density Functional Theory. J. Phys. Chem. C.

[53] Shannon R.D. (1976). Revised Effective Ionic-Radii and Systematic Studies of Interatomic Distances in Halides and Chacogenides. Acta Cryst..

[54] Tokura Y., Tomioka Y. (1999). Colossal Magnetoresistive Manganites. J. Magn. Magn. Mater..

[55] Mickevicius S., Grebinskij S., Bondarenka V., Vengalis B., Sliuziene K., Orlowski B.A., Osinniy V., Drube W. (2006). Investigation of Epitaxial LaNiO_3-X_ Thin Films by High-Energy XPS. J. Alloys Compd..

[56] Stojanovic M., Haverkamp R.G., Mims C.A., Moudallal H., Jacobson A.J. (1997). Synthesis and Characterization of LaCr_1-x_Ni_x_O_3_ Perovskite Oxide Catalysts. J. Catal..

[57] Arca E., Kehoe A.B., Veal T.D., Shmeliov A., Scanlon D.O., Downing C., Daly D., Mullarkey D., Shvets I.V., Nicolosi V. (2017). Valence Band Modification of Cr_2_O_3_ by Ni-Doping: Creating a High Figure of Merit P-Type Tco. J. Mater. Chem. C.

[58] An Z., Zhuo Y., Xu C., Chen C. (2014). Influence of the TiO_2_ Crystalline Phase of MnO_x_/TiO_2_ Catalysts for No Oxidation. Chin. J. Catal..

[59] Ichimura K., Inoue Y., Yasumori I. (1980). Catalysis by Mixed Oxide Perovskites. I. Hydrogenolysis of Ethylene and Ethane on LaCoO_3_. Bull. Chem. SOC. Jpn..

[60] Yamazoe N., Teraoka Y., Seiyama T. (1981). TPD and XPS Study on Thermal Behavior of Absorbed Oxygen in La_1-x_Sr_x_CoO_3_. Chem. Lett..

[61] Tabata K., Matsumoto I., Kohiki S. (1987). Surface Characterization and Catalytic Properties of La_1-x_Sr_x_CoO_3_. J. Mater. Sci..

[62] Tabata K., Matsumoto I., Kohiki S. (1987). Effect of Thermal Treatment on Catalytic Properties of La_0.9_Ce_0.1_CoO_3_. J. Mater. Sci..

[63] Tejuca L.G., Fierro J.L.G., Tascón J.M.D. (1989). Structure and Reactivity of Perovskite-Type Oxides. Adv. Catal..

[64] Richiter L., Bader S.D., Brodsky M.B. (1980). Ultraviolet, X-Ray-Photoelectron, and Electron-Energy-Loss Spectroscopy Studies of LaCoO_3_ and Oxygen Chemisorbed on LaCoO_3_. Phys. Rev. B.

[65] Dupin J.C., Gonbeau D., Vinatier P., Levasseur A. (2000). Systematic XPS Studies of Metal Oxides, Hydroxides and Peroxides. Phys. Chem. Chem. Phys..

[66] Kitamura M., Ohkubo I., Matsunami M., Horiba K., Kumigashira H., Matsumoto Y., Koinuma H., Oshima M. (2009). Electronic Structure Characterization of La_2_NiMnO_6_ Epitaxial Thin Films Using Synchrotron-Radiation Photoelectron Spectroscopy and Optical Spectroscopy. Appl. Phys. Lett..

[67] Mohanty S., Choudhary R.N.P., Padhee R., Parida B.N. (2014). Dielectric and Impedance Spectroscopy of BiFeO_3_–NaTaO_3_ Multiferroics. Ceram. Int..

[68] Cross L.E. (1994). Relaxor Ferroelectrics: An Review. Ferroelectrics.

[69] Yang W.Z., Liu X.Q., Zhao H.J., Lin Y.Q., Chen X.M. (2012). Structure, Magnetic, and Dielectric Characteristics of Ln_2_NiMnO_6_ (Ln = Nd and Sm) Ceramics. J. Appl. Phys..

[70] Rivera I., Kumar A., Ortega N., Katiyar R.S., Lushnikov S. (2009). Divide Line between Relaxor, Diffused Ferroelectric, Ferroelectric and Dielectric. Solid State Commun..

[71] Glass A.M. (1968). Dielectric, Thermal, and Pyroelectric Properties of Ferroelectric LiTaO_3_. Phys. Rev..

[72] Uchino K., Nomura S. (1982). Critical Exponents of the Dielectric Constants in Diffused Phase Transition Crystals. Ferroelectr. Lett. Sect..

[73] Chen X., Chen J., Huang G., Ma D., Zhou L.H. (2015). Relaxor Behaviour and Dielectric Properties of Bi(Zn_2/3_Nb_1/3_)O_3_-Modified BaTiO_3_ ceramics. J. Electron. Mater..

[74] Bensemma N., Taïbi K. (2014). Relaxor Behaviour in Lead-Free Ba(Ti_1-x_Sc_x/2_Nb_x/2_)O_3_ Ceramics. J. Asian Ceram. Soc..

[75] Reddy M.P., Shakoor R.A., Mohamed A.M.A. (2016). Structural and Magnetic Studies of La_2_BMnO_6_ (B=Ni and Co) Nanoparticles Prepared by Microwave Sintering Approach. Mater. Chem. Phys..

[76] Kittel C. (2004). Introduction to Solid State Physics.

[77] Taguchi H. (1997). Relationship between Crystal Structure and Electrical Properties of Nd(Cr_1-X_Fe_X)_O_3_. J. Solid State Chem..

[78] Coey J.M.D. (2010). Magnetism and Magnetic Materials.

[79] Choudhary N., Verma M.K., Sharma N.D., Sharma S., Singh D. (2020). Correlation between Magnetic and Transport Properties of Rare Earth Doped Perovskite Manganites La_0.6_R_0.1_Ca_0.3_MnO_3_ (R = La, Nd, Sm, Gd, and Dy) Synthesized by Pechini Process. Mater. Chem. Phys..

[80] Tauc J., Grigorov R., Vancu A. (1966). Optical Properties and Electronic Structure of Amorphous Germanium. Phys. Status Solidi.

[81] Moritomo Y., Shimamoto N., Xu S., Machida A., Nishibori E., Takata M., Sakata M., Nakamura A. (2001). Effects of B-Site Disorder in Sr_2_FeMoO_6_ with Double Perovskite Structure. Jpn. J. Appl. Phys..

[82] Arima T., Tokura Y., Torrance J.B. (1993). Variation of Optical Gaps in Perovskite-Type 3d Transition-Metal Oxides. Phys. Rev. B.

[83] Hissariya R., Babu S., Ram S., Mishra S.K. (2021). Spin-up conversion, exchange-interactions, and tailored magnetic properties in core-shell La_2_NiMnO_6_ of small crystallites. Nanotechnology..

[84] Tiwary S., Kuila S., Sahoo M.R., Barik A., Babu P.D., Vishwakarma P.N. (2018). Magnetoelectricity in La_2_NiMnO_6_ and its PVDF impregnated derivative. J. Appl. Phys..

