# Supplementary material for: Unraveling the Structural, Dielectric, Magnetic, and Optical Characteristics of Nanostructured La2NiMnO6 Double Perovskites
Source: Nanomaterials (Basel). 2022 Mar 16;12(6):979. doi: 10.3390/nano12060979 (PMC8950916; doi:10.3390/nano12060979)
Supplement: Supplementary file 1 [file nanomaterials-12-00979-s001.zip › nanomaterials-1584444-supplementary.pdf]

Electronic Supplementary Material (ESI) for Nanomaterials.

Supporting Information for

Unraveling the Structural, Dielectric, Magnetic, and Optical Characteristics of Nanostructured La_2NiMnO_6 Double Perovskites

Kang Yi [1], QingKai Tang [1], Zhiwei Wu [1], Xinhua Zhu [1,*]

National Laboratory of Solid State Microstructures, School of Physics, Nanjing University, Nanjing 210093, China; 15255458807@163.com (K.Y.); tangqinkai@126.com (Q.T.); mg20220132@smail.nju.edu (Z.W.)

* Correspondence: xhzhu@nju.edu.cn

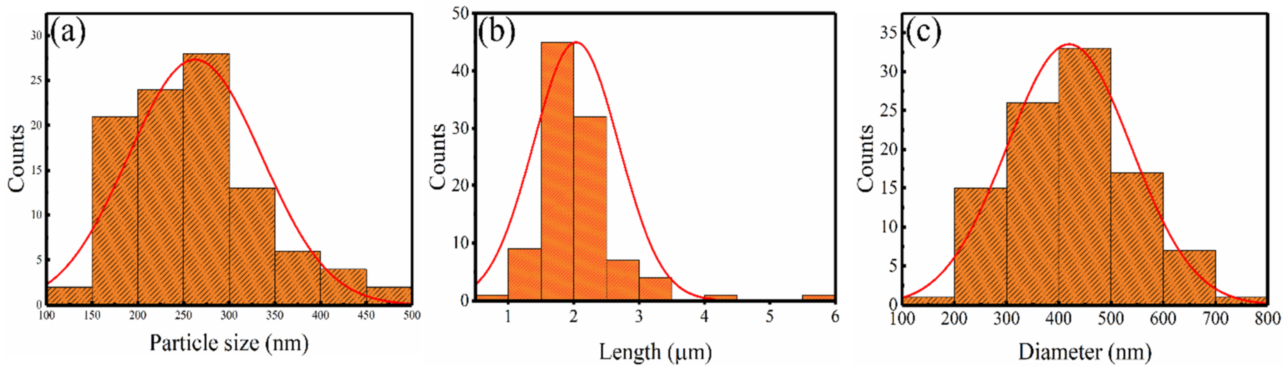

Figure S1. (a) Histogram of LNMO NPs size distribution. (b) and (c) Histograms of the distributions of the length and diameter of the LNMO NRs.

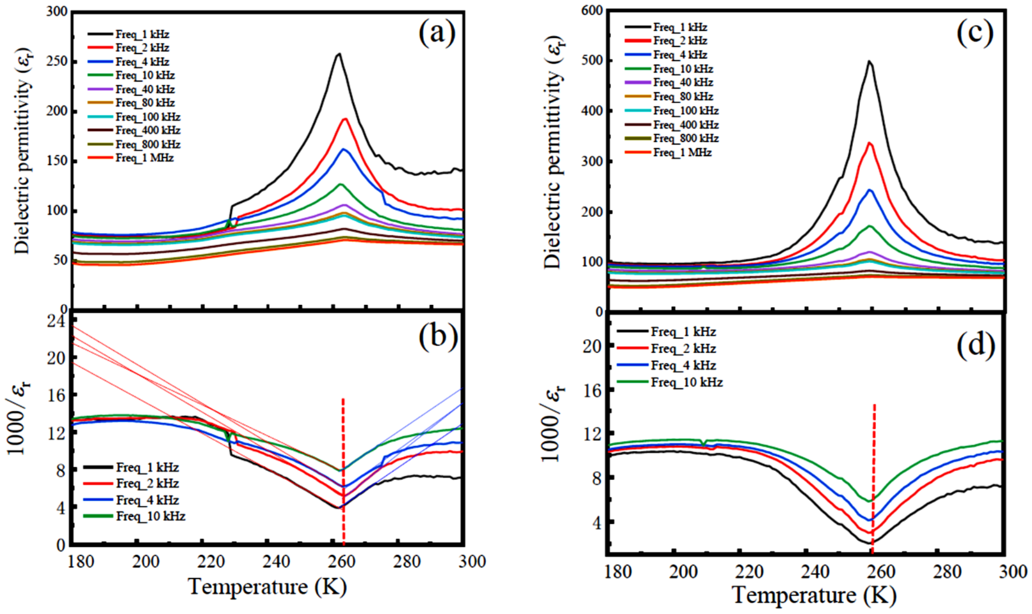

Figure S2. Temperature dependent dielectric permittivity (ε_r) and its reciprocal ($1000/\varepsilon_r$) of the LNMO ceramics prepared from (a,b) NPs and (c,d) NRs.

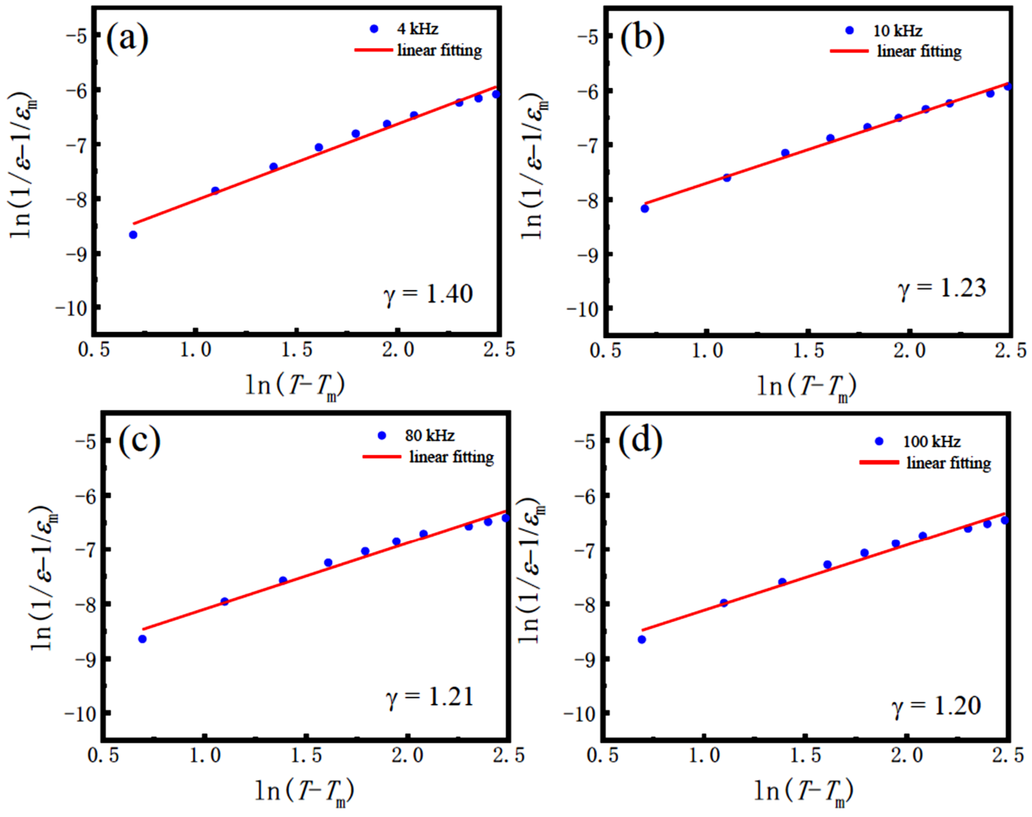

Figure S3. Plots of $\ln\left(\frac{1}{\varepsilon_r} - \frac{1}{\varepsilon_m}\right)$ as a function of $\ln\left(T - T_m\right)$ for the LNMO ceramics prepared from NPs measured at (a) 4 kHz, (b) 10 kHz, (c) 80 kHz, and (d) 100 kHz.

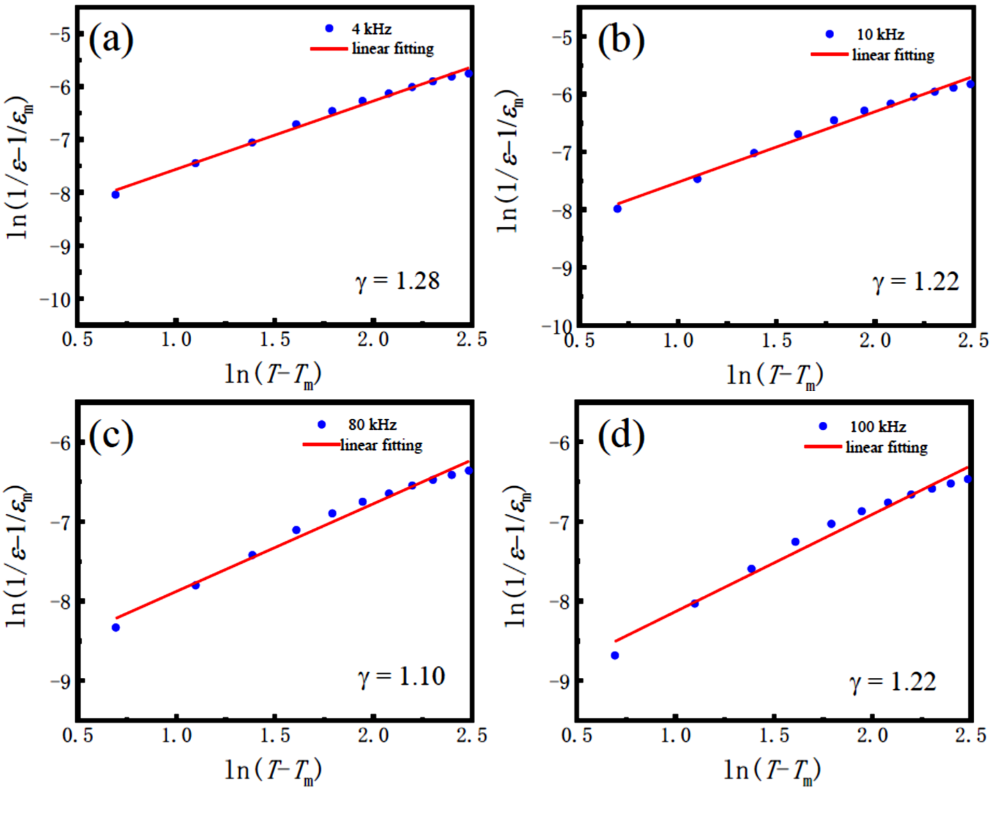

Figure S4. Plots of $\ln\left(\frac{1}{\varepsilon_r} - \frac{1}{\varepsilon_m}\right)$ as a function of $\ln\left(T - T_m\right)$ for the LNMO ceramics prepared from NRs measured at (a) 4 kHz, (b) 10 kHz, (c) 80 kHz, and (d) 100 kHz.

Table S1. Comparison between the magnetic data and Curie-Weiss fitting parameters of the as-synthesized LNMO samples (nanoparticles (NPs), nanorods (NRs), thin films, and bulk) and that reported in the recent literatures.

**Synthesis Method**	**Forms**	T_c **(K)**	θ_p **(K)**	C **(emu·K/mol)**	μ_{eff} **(μ_B)**	μ_{cal} **(μ_B)**	M_{Sexp} **(μ_B/f.u.)**	M_{Scal} **(μ_B/f.u.)**	**Ref.**
hydrothermal method	NPs	252.0	257.0	3.69	5.53	5.51	6.20	6.18	this work
	NRs	232.0	235.8	4.48	5.99	5.35	5.68	5.99	
hydrothermal method	NRs	178.0		8.86	8.42	5.85	6.30	6.30	[46]
Solid-state reaction	Bulk	280.0	274.0	3.84	5.5	5.92	4.96	5.00	[3]
	NPs	260				6.16	4.70		[83]
Sol-gel method	NPs	196.0	232.0				4.50		[30]
	NPs	280.0	283.0	3.66	5.41	4.79	4.95		[7]
	NPs	275.0					3.40		[84]
	NPs	266.0			5.76	4.15	3.16		[26]
Co-precipitation route	NPs	171.0	177	1.90	3.31		3.97		[40]
pulsed-laser deposition	Thin-films	265					4.00		[12]
microwave sintering technique	NPs	270					3.74		[75]
